# The Most Popular Commercial Weight Management Apps in the Chinese App Store: Analysis of Quality, Features, and Behavior Change Techniques

**DOI:** 10.2196/50226

**Published:** 2023-11-24

**Authors:** Lan Geng, Genyan Jiang, Lingling Yu, Yueming Xu, Wei Huang, Zhiqi Chen, Xiaoyan Qi, Ting Zhang, Mei Zhao

**Affiliations:** 1 School of Nursing Anhui Medical University Hefei China

**Keywords:** quality, mobile health, mobile apps, weight loss, weight management, apps, MARS, China

## Abstract

**Background:**

Many smartphone apps designed to assist individuals in managing their weight are accessible, but the assessment of app quality and features has predominantly taken place in Western countries. Nevertheless, there is a scarcity of research evaluating weight management apps in China, which highlights the need for further investigation in this area.

**Objective:**

This study aims to conduct a comprehensive search for the most popular commercial Chinese smartphone apps focused on weight management and assess their quality, behavior change techniques (BCTs), and content-related features using appropriate evaluation scales. Additionally, the study sought to investigate the associations between the quality of various domains within weight management apps and the number of incorporated BCTs and app features.

**Methods:**

In April 2023, data on weight management apps from the iOS and Android app stores were downloaded from the Qimai Data platform. Subsequently, a total of 35 weight management apps were subjected to screening and analysis by 2 researchers. The features and quality of the apps were independently assessed by 6 professionals specializing in nutrition management and health behavioral change using the Mobile Application Rating Scale (MARS). Two registered dietitians, who had experience in app development and coding BCTs, applied the established 26-item BCT taxonomy to verify the presence of BCTs. Mean (SD) scores and their distributions were calculated for each section and item. Spearman correlations were used to assess the relationship between an app’s quality and its technical features, as well as the number of incorporated BCTs.

**Results:**

The data set included a total of 35 apps, with 8 available in the Android Store, 10 in the Apple Store, and 17 in both. The overall quality, with a mean MARS score of 3.44 (SD 0.44), showed that functionality was the highest scoring domain (mean 4.18, SD 0.37), followed by aesthetics (mean 3.43, SD 0.42), engagement (mean 3.26, SD 0.64), and information (mean 2.91, SD 0.52), which had the lowest score. The mean number of BCTs in the analyzed apps was 9.17 (range 2-18 BCTs/app). The most common BCTs were “prompt review of behavioral goals” and “provide instruction,” present in 31 apps (89%). This was followed by “prompt self-monitoring of behavior” in 30 apps (86%), “prompt specific goal setting” in 29 apps (83%), and “provide feedback on performance” in 27 apps (77%). The most prevalent features in the analyzed apps were the need for web access (35/35, 100%), monitoring/tracking (30/35, 86%), goal setting (29/35, 83%), and sending alerts (28/35, 80%). The study also revealed strong positive correlations among the number of BCTs incorporated, app quality, and app features. This suggests that apps with a higher number of BCTs tend to have better overall quality and more features.

**Conclusions:**

The study found that the overall quality of weight management apps in China is moderate, with a particular weakness in the quality of information provided. The most prevalent BCTs in these apps were reviewing behavioral goals, providing guidance, self-monitoring of behavior, goal setting, and offering performance feedback. The most common features were the need for web access, monitoring and tracking, goal setting, and sending alerts. Notably, higher-quality weight management apps in China tended to incorporate more BCTs and features. These findings can be valuable for developers looking to improve weight management apps and enhance their potential to drive behavioral change in weight management.

## Introduction

### Background

Obesity is becoming increasingly prevalent on a global scale, and this poses a significant challenge to public health worldwide. One of the primary contributing factors to this concerning trend is the expansion of global free trade, coupled with the economic growth and urbanization experienced by many low- and middle-income countries. These changes have given rise to environments that promote unhealthy dietary habits and a reduction in physical activity (PA) [1]. By the year 2025, it is anticipated that the worldwide prevalence of obesity will reach 18% among men and exceed 21% among women. Additionally, the prevalence of severe obesity is projected to surpass 6% for men and 9% for women [2]. Between 1980 and 2015, excess body weight has been linked to more than 4 million deaths worldwide [3]. Recent statistics indicate that over 50% of adults in China are overweight or obese, establishing China as the country with the highest number of individuals affected by overweight or obesity in the world [4]. Newly published studies have forecasted that by 2030, the combined prevalence of overweight and obesity among Chinese adults aged 18 years and older will reach 70.5% [5]. China’s rate of overweight and obesity is significantly higher than that of the Middle East (21.17%) [6], France (37.3%), Portugal (43.3%), and Italy (44.0%) [7]. However, it is slightly lower than the rates in Romania (58.8%), Latvia (57.3%), and Bulgaria (56.9%) [7]. Nonetheless, owing to its vast population, China has the highest count of individuals with overweight or obesity globally [4]. This concerning statistic is expected to escalate to 810.65 million by 2030 [5], underscoring the gravity of the national health situation. Obesity has emerged as the leading cause of poor health, surpassing malnutrition and infectious diseases [8]. Excess body weight, particularly obesity, is the primary lifestyle-related risk factor for premature death. This increases the risk of morbidity and mortality from various diseases and conditions, such as cardiovascular disease, type 2 diabetes mellitus, nonalcoholic fatty liver disease, and certain types of cancer [8]. Hence, it holds significant importance and urgency to focus on behavioral interventions for individuals with overweight or obesity and bolster weight management. For decades, many countries have implemented policies aimed at promoting healthy lifestyles to prevent obesity [1], but these efforts have not succeeded in curbing the prevalence of obesity. At present, the scientific community is placing a priority on researching interventions that are accessible, cost-effective, and customized to individual needs. The goal of these interventions is to facilitate the translation of health knowledge into practical health behaviors and skills, a task that poses a significant challenge.

In the digital age, smartphones serve a purpose beyond making phone calls; they are now widely integrated into various aspects of life, including transportation, shopping, and social entertainment. Consequently, they have a profound impact on both people’s work and personal lives. In China, smartphone ownership has witnessed exponential growth over the past decade. According to a statistical report on the development of the Chinese internet, by December 2020, there were 989 million Chinese internet users, of which a staggering 986 million (99.7%) used smartphones to access the internet [9].The widespread adoption of smartphones has spurred the rapid growth of the mobile app market. In an environment where the country and society actively promote national health, people’s awareness of health and their expectations for health standards continue to rise. This has led to increased demands for personal health management, the development of healthy behaviors, and opportunities for healthy social engagement, among other aspects.According to available data, there are 660 million mobile health users in China [10]. Among these users, weight management is one of the most prominent demands, representing 41.9% of all requests [11]. Compared with traditional weight management methods, mobile apps are more cost-effective [12] and have the advantage of transcending time and space constraints. They enable users to monitor their personal health status and access essential health information whenever and wherever they desire. The convenience and practicality of weight management apps have made them highly popular among users, establishing them as a vital tool for effective weight management.

The weight management app market in China is experiencing rapid growth. Nevertheless, the content and quality of the available apps remain somewhat unclear. While app stores offer star ratings, user comments, and installation statistics, there is a notable absence of quality indicators specifically tailored to weight management apps. Furthermore, star ratings and comments can sometimes include inaccurate or subjective feedback, potentially causing confusion among users [13]. In the pursuit of offering users higher-quality mobile apps, an expert team has devised the Mobile Application Rating Scale (MARS). This scale serves as a straightforward, objective, and dependable tool for researchers, developers, and health professionals to assess app quality [14]. Since its inception in 2015, the MARS has been translated into multiple languages [15-17] and used to assess a wide range of mobile apps [18-26]. It comprises 23 items organized into distinct sections, covering engagement, functionality, aesthetics, information quality, and subjective quality [14]. Furthermore, mobile health (mHealth) apps that incorporate established behavior change techniques (BCTs) rooted in behavior change theories and designed to offer practical principles for altering determinants of behavior have the potential to facilitate behavioral change [27]. Nevertheless, not all commercially available weight management apps include BCTs [28]. Previous studies have evaluated English weight management apps from multiple angles, encompassing quality, features, and the presence of BCTs [28-30], but these studies cannot offer a comprehensive assessment of the quality of the currently available weight management apps in China.

### Objectives

To fill this void, the objectives of this study were 4-fold: (1) to conduct a systematic assessment of the quality of well-received weight management apps; (2) to determine the count of BCTs and content-related features within these apps; (3) to evaluate the associations between various aspects of app quality and the inclusion of BCTs in these apps; and (4) to explore the connections between app quality and the number of app features.

## Methods

### Search Strategy

Qimai Data (Beijing Qimai Technology Co., Ltd.) is a specialized platform for analyzing mobile app data in China. It encompasses both the App Store (Apple Inc.) and Google Play Store (Alphabet Inc.) platforms, delivering comprehensive data insights for the iOS and Android app markets [31]. This platform constituted the primary data source for our study. In April 2023, 2 reviewers (LG and GYJ), 1 registered dietitian and 1 postgraduate in nutrition, performed a systematic search of mobile apps associated with weight management. This search encompassed 9 Chinese language mobile app stores and was facilitated using Qimai Data. The stores included the Apple iTunes Store for iOS and the Android system stores, including Tencent My App, Huawei, Xiaomi, VIVO, OPPO, Meizu, 360 Mobile Assistant and Baidu Mobile Assistant. The search was conducted using the keywords obesity, slimming, weight loss, weight, and fitness. Each keyword was entered into the general search bar of the 9 app stores mentioned above. Data extracted from Qimai Data were app names, subtitles, developers, rating scores, update time stamps, the number of ratings, and cumulative downloads. The inclusion or exclusion of the apps was documented following the PRISMA (Preferred Reporting Items for Systematic Reviews and Meta-Analyses) flow diagram [32].

### App Selection

The app filtering process involved several steps. Initially, information about weight management apps was retrieved and downloaded from the Qimai Data platform, with duplicate apps removed. Second, 2 reviewers (LG and GYJ) assessed app eligibility based on the app’s title and introduction. An app was deemed eligible for inclusion in the study only if it was relevant to weight management, available in the Chinese language, designed for use by non–health care professionals, and had been updated within the preceding 2 years

Third, apps were excluded if they fell into the following categories: (1) they primarily addressed health aspects unrelated to weight management or related behaviors [28], such as smoking or sleeping; (2) they were purely focused on sports and fitness [28]; (3) they had a user rating below 4 on a scale ranging from 1 (inadequate) to 5 (excellent) [28]; and (4) they had accumulated less than 10,000 downloads.

**Figure 1:**
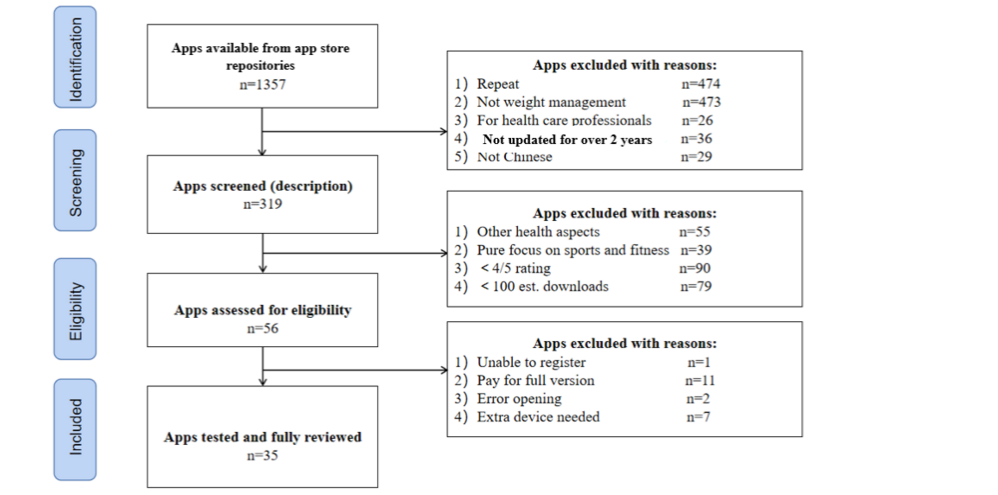
Flow diagram of app search and selection.

Finally, apps requiring external devices, such as scales or wrist straps for proper functioning, and those that necessitated the purchase of a full version were excluded. We also removed apps that did not allow for registration or displayed errors upon opening. The app selection process is outlined in Figure 1. Following 4 rounds of screening, a collection of apps was identified and made ready for download and evaluation. This selection and evaluation process took place from April 2023 to May 2023.

### Data Extraction

A team consisting of 6 raters (LG, GYJ, LLY, YMX, MZ, and TZ) downloaded the chosen apps and conducted independent assessments of their quality and features. The team comprised individuals with backgrounds in nursing and graduate students with research interests in clinical nutrition. Among the 6 reviewers, 2 were registered dietitians, and 4 were graduate nursing students. All members of the team possessed experience in nutrition management and health behavioral change, with 2 registered nutritionists having additional expertise in coding BCTs and app development. The raters used a variety of devices, including OPPO R15X, Huawei nova 11 Ultra, Redmi Note 8 Pro (Xiaomi), and OPPO R17, to download and evaluate apps from the Tencent App Store. For apps from the Apple App Store, iPhone 13 running iOS 15.6 and iPhone 12 running iOS 16.1.1 were used for downloading and testing. Each app was tested for 1 day. For all the apps included in the study, the following app characteristics were collected: app name; developer; version; date of the most recent update; average user rating (with a minimum of 4+); cumulative installation count; presence of BCTs; focus areas according to the MARS (such as enhancing happiness/well-being, behavioral change, entertainment, physical health); MARS theoretical underpinnings/strategies (including assessment, goal setting, information/education, advice/tips/strategies/skills training); and MARS technical features (eg, sharing capabilities, password protection, reminder notifications).

### App Quality and Features Assessment

Six independent evaluators assessed app quality and features using the MARS [14]. Prior studies have demonstrated that the scale exhibits strong internal consistency and interrater reliability [33]. The MARS comprises 19 items, categorized into 4 dimensions: engagement (5 items, including entertainment, interest, customization, interactivity, and target group), functionality (4 items, encompassing performance, ease of use, navigation, and gestural design), aesthetics (3 items, covering layout, graphics, and visual appeal), and information (7 items, involving accuracy of app description, goals, quality of information, quantity of information, visual information, credibility, and evidence base). Each item was rated on a 5-point scale (1=inadequate; 2=poor; 3=acceptable; 4=good; and 5=excellent). The overall MARS score is calculated as the average of the 4 dimensions, providing an assessment of the app’s overall quality. The MARS score ranges from 1 to 5, with higher scores indicating better app quality. A minimum acceptable score of 3.0 has been previously established as a cutoff point [34].

Beyond the 4 objective quality scales, the MARS also includes assessments of subjective app quality, which encompasses 4 items: recommendation, frequency of use, willingness to pay, and overall star rating. Additionally, it evaluates the likelihood of behavioral impact in weight management, considering 6 items: awareness, knowledge, attitudes, intention to change, help-seeking, and behavioral change. The subjective quality and likelihood of behavioral impact in weight management items were evaluated and scored independently.

### Presence of BCTs Assessment

The presence of BCTs was evaluated by 2 registered dietitians using the taxonomy of BCTs [27]. Both registered dietitians possess experience in providing health behavioral change support, nutritional management, coding BCTs, and app development. The taxonomy of BCTs was developed by Abraham and Michie [35], and it was used to assess the presence or absence of BCTs in the included apps. This taxonomy comprises 26 BCTs, which can be used to gauge the effectiveness of behavior change interventions. It has also been used in previous studies to assess the presence of BCTs, similar to the approach taken in our study [36]. In this study, the 26 BCTs from the BCT taxonomy were used to assess the included apps, with 0 indicating absence and 1 indicating presence.

### Quality Control

Initially, the 6 raters underwent training using the handbook to ensure their correct usage of the MARS and successfully completed a pilot test [14]. Before rating the apps included in the study, the raters randomly selected 6 weight management apps that were not part of the study, assessed them using the MARS, and discussed their findings. This step was taken to ensure that the raters were acquainted with the MARS program and the evaluation process. Any points of disagreement were deliberated until a consensus was ultimately reached. Subsequently, for all 35 eligible apps, the 6 raters initially downloaded and utilized all the functions of the apps on their own smartphones for 1 day to acquaint themselves with the apps. Lastly, the raters individually conducted the formal assessments of the 35 eligible apps using the MARS. For those apps that the raters could not access on their own smartphones due to compatibility issues, such as rater A with an Android smartphone system being unable to use an app on rater B’s Apple smartphone system, the research team organized regular formal discussion meetings. On the first day, the raters swapped smartphones to become acquainted with the app interfaces and functions on each other’s phones. Each app was used for a minimum of 20 minutes. On the second day, the app was used to gain further familiarity. On the third day, the app was used and formally evaluated using the MARS.

### Statistical Analysis

Data analysis was conducted using SPSS version 26.0 (IBM Corp.). Descriptive statistics were applied to app quality and BCT data, and continuous variables were presented as mean and SD. If the data distribution was skewed, the median and IQR were reported. The interrater reliability between the raters for MARS scores and the number of BCTs present in the apps were assessed using 2-way mixed effects intraclass correlation coefficients. Agreement scores below 0.5 were considered indicative of poor agreement, while those within the range of 0.5-0.75 represented moderate reliability. Scores falling between 0.75 and 0.9 were indicative of good reliability, and scores exceeding 0.9 demonstrated excellent reliability [37]. The internal consistency of the MARS was evaluated using Cronbach α, while the internal consistency of BCTs was assessed using the Kuder-Richardson index 20 (KR-20). Spearman correlations were used to investigate the relationships between app quality, the number of technical app features, and the number of BCTs integrated into the apps. A correlation coefficient of >0.7 indicates a strong correlation, while a range of 0.3-0.7 suggests a moderate correlation, and <0.3 indicates a low correlation [38].

### Ethical Considerations

This study was approved by the Ethics Committee of Anhui Medical University under the ethical number 84230017.

## Results

### Identification of Apps

A search was conducted in the Chinese app market for weight management apps available for iOS and Android systems, yielding 899 and 458 apps, respectively. The apps were subsequently screened according to the specified inclusion and exclusion criteria, ultimately resulting in the identification of 35 independent apps for analysis. Figure 1 presents an overview of the selection process and delineates the reasons for exclusion.

### Characteristics and Features of the Selected Apps

Table 1 displays the characteristics and features of the apps included in this study. Of the 35 apps included, 10 were exclusive to iOS, 8 were exclusive to Android, and 17 apps were available on both platforms. Several apps were entirely free (12/35, 34%), but a larger number required payment for advanced services (23/35, 66%). The health behaviors targeted by the apps encompassed diet (28/35, 80%) and PA (21/35, 60%), with 16 apps addressing both of these behaviors. The majority of the apps necessitate a log-in (20/35, 57%), send reminders (28/35, 80%), and require internet access (35/35, 100%). Furthermore, they offer functions such as information/education (20/35, 57%), monitoring/tracking (30/35, 86%), goal setting (29/35, 83%), and advice/tips/strategies/skill training (27/35, 77%). Some apps provide the option for sharing (12/35, 34%), have an integrated app community (16/35, 46%), and offer password protection (14/35, 40%).

**Table 1:** Descriptive data of the included apps (N=35).

Descriptive data			Values						
			n (%)		Mean (SD)		Median (IQR)		Range (min-max)
**App store**									
	Android	8 (23)		N/A^a^		N/A		N/A	
	Apple	10 (29)		N/A		N/A		N/A	
	Android and Apple	17 (49)		N/A		N/A		N/A	
**Cost**									
	Free	12 (34)		N/A		N/A		N/A	
	Paid (costs in RMB ￥^b^)	23 (66)		29.41 (26.81)		16.50 (47.20)		75.00 (3.00-78.00)	
**User rating**									
	Average rating (4+)	35 (100)		4.63 (0.29)		4.80 (0.30)		1.00 (4.00-5.00)	
	The average number of user ratings (count)	35 (100)		114,684 (519,244)		2208 (17,548)		3,050,463 (25-3,050,488)	
**Health behavior**									
	Diet	28 (80)		N/A		N/A		N/A	
	Physical activity	21 (60)		N/A		N/A		N/A	
	Diet and physical activity	16 (46)		N/A		N/A		N/A	
**Age group**									
	Children (≤12 years)	22 (63)		N/A		N/A		N/A	
	Adolescents (13-17 years)	30 (86)		N/A		N/A		N/A	
	Young adults (18-25 years)	35 (100)		N/A		N/A		N/A	
	Adults (>25 years)	35 (100)		N/A		N/A		N/A	
Number of app features (0-10)			N/A		6.00 (2.55)		6.00 (4)		8.00 (2.00-10.00)
**App features**									
	Allows sharing	12 (34)		N/A		N/A		N/A	
	Has an app community	16 (46)		N/A		N/A		N/A	
	Allows password protection	14 (40)		N/A		N/A		N/A	
	Requires log-in	20 (57)		N/A		N/A		N/A	
	Sends reminders	28 (80)		N/A		N/A		N/A	
	Needs web access to function	35 (100)		N/A		N/A		N/A	
	Information/education	20 (57)		N/A		N/A		N/A	
	Monitoring/tracking	30 (86)		N/A		N/A		N/A	
	Goal setting	29 (83)		N/A		N/A		N/A	
	Advice/tips/strategies/skills training	27 (77)		N/A		N/A		N/A	

^a^N/A: not applicable.

^b^RMB ¥1 = US $0.14.

### App Quality

Table 2 presents the overall MARS scores and scores for each domain of the assessed apps. The interrater reliability for app quality assessment was high, with an intraclass correlation coefficient of 0.85 (95% CI 0.75-0.92) for the overall MARS score, 0.87 (95% CI 0.79-0.93) for engagement, 0.77 (95% CI 0.61-0.87) for functionality, 0.72 (95% CI 0.54-0.84) for aesthetics, 0.90 (95% CI 0.84-0.94) for information, 0.79 (95% CI 0.61-0.89) for subjective app quality, and 0.89 (95% CI 0.82-0.94) for app-specific quality. The internal consistency of the total MARS score and scores for each domain were considered excellent (Cronbach α>.70; Table 2).

The average total MARS score was 3.44 (out of 5), with scores ranging from 2.82 to 4.40, signifying a moderate overall quality. Functionality received the highest domain score, followed by aesthetics, engagement, and information. The average subjective quality score was 2.38, suggesting a low quality of the evaluated apps in terms of subjective assessments. The average app-specific quality score was 2.50, indicating a low quality concerning the potential to change awareness, knowledge, attitudes, intentions, help-seeking, and behavioral change (Table 2).

Table 3 presents the app quality scores and BCT scores separately for the 2 app platforms. The findings reveal that there were no significant differences in the scores for engagement (*P*=.93), functionality (*P*=.76), aesthetics (*P*=.77), information (*P*=.66), the overall MARS score (*P*=.85), subjective quality score (*P*=.88), app-specific quality score (*P*=.87), and BCT score between the 2 app platforms (*P*=.94).

**Table 2:** App quality and behavior change technique assessment scores.

Mobile Application Rating Scale domain	Mean (SD)	Median (range)	Cronbach α
Engagement (5 items)	3.26 (0.64)	3.20 (2.10-4.30)	.84
Functionality (4 items)	4.18 (0.37)	4.11 (3.70-4.90)	.74
Aesthetics (3 items)	3.43 (0.42)	3.42 (2.60-4.30)	.82
Information (7 items)	2.91 (0.52)	2.90 (2.10-4.30)	.83
App quality (overall mean)	3.44 (0.44)	3.34 (2.82-4.40)	.76
App subjective quality (4 items)	2.38 (0.75)	2.10 (1.30-4.30)	.82
App-specific quality (6 items)	2.50 (0.77)	2.20 (1.30-4.40)	.71
Number of behavior change techniques (26 items)	9.17 (4.07)	9.00 (2.00-18.0)	.87^a^

^a^The Kuder-Richardson index responses for all Mobile Application Rating Scale domains ranged from 1 to 5, with higher scores indicating a higher degree of app quality. The *P* value in the Kruskal-Wallis test between the 4 main domains of the Mobile Application Rating Scale was <.001.

**Table 3:** Scores for app quality and behavior change technique assessments, categorized by app platform.^a^

Mobile Application Rating Scale domain	App platform		*P* value
	Android (n=25), mean (SD)	Apple (n=27), mean (SD)	
Engagement	3.35 (0.89)	3.33 (0.88)	.93
Functionality	4.49 (0.31)	4.34 (0.49)	.76
Aesthetics	3.8 (0.42)	3.66 (0.58)	.77
Information	3.02 (0.83)	3.16 (0.63)	.66
App quality (overall mean)	3.67 (0.54)	3.61 (0.56)	.85
App subjective quality	2.71 (1.01)	2.65 (1.03)	.88
App-specific quality	2.73 (1.01)	2.83 (0.89)	.87
Number of behavior change techniques	9.24 (4.62)	9.85 (4.27)	.94

^a^All item responses in the Mobile Application Rating Scale ranged from 1 to 5, with higher scores signifying a higher level of app quality. Differences in scores and the number of behavior change techniques between platforms were evaluated using Mann-Whitney tests.

### Presence of BCTs

**Figure 2:**
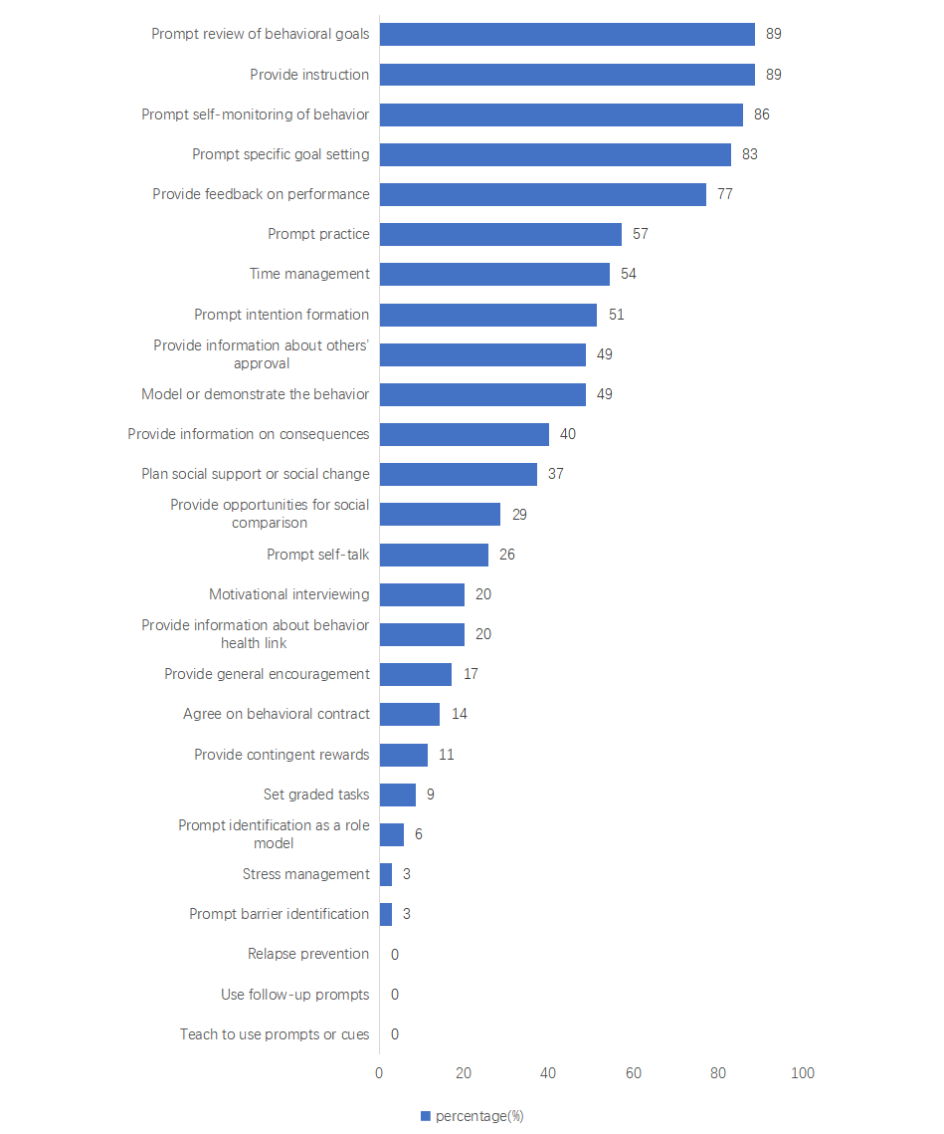
Presence of individual behavior change techniques in the analyzed apps..

The internal consistency in the evaluation of BCTs was likewise determined to be excellent, with the Kuder-Richardson value exceeding 0.70 (Table 2). The mean number of BCTs in the analyzed apps was 9.17 (range 2-18), indicating a moderate presence of BCTs in the evaluated apps. All apps contained a minimum of 2 BCTs, with 1 app (Boohee Health) having the highest recorded count of 18 BCTs. Figure 2 illustrates the proportion of apps that were evaluated to have the presence of BCTs. The most frequently observed BCTs were “prompt review of behavioral goals” and “provide instruction” (31/35, 89%), followed by “prompt self-monitoring of behavior” (30/35, 86%), “prompt-specific goal setting” (29/35, 83%), and “provide feedback on performance” (27/35, 77%). Three BCTs, namely, “relapse prevention,” “use follow-up prompts,” and “teach to use prompts or cues,” did not appear in any of the evaluated apps (Figure 2).

### Relationship Between App Quality and the Presence of BCTs/App Features

The number of BCTs exhibited a significant positive correlation with the MARS overall score (r=0.74; *P*<.01), MARS subjective quality score (r=0.56; *P*<.01), MARS-specific quality score (r=0.81; *P*<.01), MARS engagement score (r=0.84; *P*<.01), aesthetic quality score (r=0.55; *P*<.01), and information quality score (r=0.81; *P*<.01). There was no significant correlation between the functional score of the apps and the number of BCTs (r=0.30; *P*=.08). The number of app features displayed a strong and positive correlation with the objective quality of the app, subjective quality, and each domain. Furthermore, the number of app features was positively associated with the number of BCTs (Table 4).

**Table 4:** Correlations between app quality, number of app features, and behavior change techniques.

MARS^a^ domain	Number of behavior change techniques	Number of app features
MARS engagement score	0.84^b^	0.77^b^
MARS functionality score	0.30	0.48^b^
MARS aesthetics score	0.55^b^	0.57^b^
MARS information quality score	0.81^b^	0.77^b^
MARS total score	0.74^b^	0.72^b^
App subjective quality	0.56^b^	0.49^b^
App-specific quality	0.81^b^	0.69^b^
Number of app features	0.69^b^	1

^a^MARS: Mobile App Rating Scale.

^b^*P*<.01.

## Discussion

### Principal Findings

After conducting a comprehensive search and screening of over 1000 apps, 35 were chosen for an in-depth evaluation in this study. This selection provided a snapshot of publicly available mobile apps for weight management in China. Through a comprehensive and systematic review, the study revealed that the overall quality of these apps was moderate, with the functionality domain achieving the highest score and the information domain attaining the lowest score based on the MARS evaluation. On average, each app included 6 features and 9.17 BCTs. The most common features were the requirement for web access to function, monitoring/tracking, goal setting, and sending reminders. The most common BCTs were prompting the review of behavioral goals, providing guidance, prompting self-monitoring of behavior, specific goal setting, and providing performance feedback. It was observed that apps with higher quality, as measured by the MARS, tended to include a greater number of features and BCTs. However, there were no significant differences in the quality or the presence of BCTs between different app platforms.

### App Features

Of the 35 apps selected, 80% (28/35) provided only diet-related information, 60% (21/35) provided only PA-related information, and 46% (16/35) offered a combination of both types of information. The primary approach to weight loss is dietary calorie restriction [39], as PA alone tends to have only a moderate effect [40]. However, combining a healthy diet with PA is more beneficial for weight loss [41]. The Chinese Nutrition Society revised the 2022 edition of dietary guidelines for Chinese residents and also proposed the recommendation to “maintain a healthy weight by not overeating and being physically active every day.” As nutrition management and exercise are 2 major user demands for digital health management in China [11], and they are also essential and effective approaches for weight management, it is crucial that, in the future, when developing high-quality weight management apps, these 2 dimensions are integrated and emphasized. Furthermore, upon analyzing the app features, it was observed that options allowing for sharing and the presence of an app community were limited. Previous studies have indicated that engagement is crucial for enhancing the health of users, and apps that provide interactive features tend to score higher in the engagement quality domain [28,42].

Hence, in this study, the absence of interactive features such as app community sharing led to low engagement scores. Health-related social interactions are the primary trend among users of mobile health apps. Users’ main demands are tracking and recording their training results, enhancing enjoyment, sharing experiences, and boosting motivation. According to a previous study [11], a significant number of users (73.4%) were interested in health apps because they provide access to quality or professional users for communication [11]. With the onset of the 5G era, the social value in mobile health apps will be further enhanced and expanded. Hence, interactivity is an essential factor for the development of high-quality apps in the future.

### App Quality

App quality was evaluated using the MARS scale, which assessed engagement, functionality, aesthetics, and information quality. We also conducted assessments for subjective quality and specific quality. The overall quality of the included apps was considered moderate, with an average MARS score of 3.44. The functionality domain received the highest score, followed by aesthetics and engagement, while the lowest-scoring domain was information quality. This finding aligns with previous studies that used MARS to evaluate the quality of health-related apps [27,43]. The high MARS functionality scores and the medium to high MARS aesthetics scores may be attributed to developers emphasizing a visually appealing and user-friendly app user experience [44]. This aligns with the concept that users typically prefer apps that are well-designed, engaging, user-friendly, and feature-rich. While these attributes attract users, they may not necessarily result in behavioral change [45].

The lowest score was observed in the information quality domain, indicating a general absence of high-quality, evidence-based content. The absence of evidence-based information could potentially expose end users to incorrect or misleading content, thereby increasing the risk of negative health outcomes among weight management users. Indeed, the credibility of the source of information and the scientific evidence presented to support the content play a significant role in establishing the trustworthiness of the app [46]. These findings indicate that developers of weight management apps should prioritize the improvement of information quality by offering clearly labeled and evidence-based scientific information to enhance the overall quality of the apps. Furthermore, government public health departments and network regulators should place a significant emphasis on ensuring the quality and safety of publicly available apps. They should also intensify their advocacy efforts aimed at promoting public health literacy and increasing awareness about the potential benefits and risks associated with the use of health apps. This approach will assist users in making informed decisions and selecting apps that are evidence based and promote their health [47].

Furthermore, the subjective quality of the analyzed apps was rated as low, with an average score of 2.38, which aligns with findings in existing studies [45,48]. One possible reason for this could be the absence of scientific evidence to substantiate the information content, leading to a low score in the information domain, which, in turn, diminishes the app’s credibility [46]. The second reason could be the lack of an adequate information/education feature, with only 57% (20/35) of apps incorporating this feature. Insufficient information/education results in a knowledge gap among users regarding weight management, which hinders the enhancement of their awareness, motivation, and behavioral change. For users, understanding the reasons for engaging in weight management is crucial, as awareness of these benefits can serve as a motivating factor for them to adopt such practices. Our findings are consistent with the Information, Motivation, Behavioral Skills model of behavioral change [49]. According to this model, individuals require 3 components to initiate behavioral action: essential information (such as comprehensive knowledge related to weight management), motivation to pursue the goal (eg, willingness to embark on weight management), and behavioral skills required to make progress toward the goal (eg, self-guidance on managing nutrition and PA). The Information, Motivation, Behavioral Skills model illustrates that information can be transformed into actions that have the potential to motivate individuals and ultimately shape their attitudes and behaviors. Furthermore, “information” is regarded as the primary prerequisite for initiating behavior [49,50]. The absence of these features could diminish the user’s subjective experience. The third reason may be the lack of interactive features such as an app community and sharing capabilities. These features allow users to document and share their experiences through photos and texts and exchange insights with other users, contributing to the enjoyment and motivation of using the app [11]. Thus, developers of weight management apps should regularly reassess the features of these apps to enhance both overall and subjective quality.

### Behavior Change Techniques Included in Apps and App Features

Among the 26 BCT classifications developed by Abraham and Michie [35], there was a significant variation in the number of BCTs included in the analyzed apps, ranging from 2 to 18. In our study, each app contained 9.17 BCTs on average. This is higher than the findings of previous research, which reported an average of 4.2 [50,51] and 7.0 [52] BCTs for apps promoting PA in adults. The number of BCTs found in this review is slightly lower than that observed in a recent study that examined the number of BCTs in apps targeting PA, where an average of 11 BCTs per app was reported [53]. The inclusion of certain PA apps, which were developed with the involvement of health care professionals, may contribute to this difference in the number of BCTs. By contrast, the weight management apps examined in this study are commercially oriented. It is worth noting that apps developed with the involvement of health care professionals tend to have a stronger scientific foundation.

According to Michie et al [54], interventions aimed at improving diet and PA efficiency were associated with 5 specific BCTs, namely, prompt intention formation, prompt self-monitoring, providing feedback on performance, prompt review of behavioral goals, and prompt specific goal setting. In this study, the most commonly utilized BCTs were goal setting, reviewing behavioral goals, providing guidance, self-monitoring, and offering performance feedback, covering 4 out of the 5 valid BCTs. The 4 most commonly identified BCTs in apps targeting weight and PA in adults are goal setting, self-monitoring, providing instruction, and performance feedback [28,51]. From a psychological perspective, as specified by control theories, setting goals, monitoring behavior, receiving feedback, and reviewing relevant goals in light of feedback are central to self-management and behavioral control [12]. The apps analyzed in this study included a review of behavioral goals in addition to the 4 most effective BCTs. It is worth noting that interventions with a higher number of BCTs tend to be more effective [55]. However, it is essential to consider that incorporating too many BCTs can potentially reduce user engagement and app validity [43]. The optimal number and combination of BCTs for improving the effectiveness of weight management remain unclear. It is important to note that the impact of individual BCTs is often relatively small. To illustrate, according to control theory, setting goals and reviewing progress based on feedback are at the core of self-management, behavior control, and ultimately, behavioral change [12]. Participants can effectively self-monitor their health and make sustainable long-term changes [56]. As a result, combining techniques related to goal setting and goal review within an app may yield greater effectiveness. This insight has motivated developers to incorporate additional BCTs into future app designs and to experiment with different combinations of BCTs to determine which ones are most appealing and effective for specific populations.

Many Chinese apps offer a tracking feature (30/35, 86%). Users can record various parameters such as their daily diet, water intake, exercise, and weight. Additionally, these apps include reminders (28/35, 80%), which help users stay on track by sending prompts for meals, hydration, and exercise. They also set up goal achievement reminders to motivate users by celebrating each successful weight loss goal. Furthermore, these apps provide a range of functions, including advice, tips, strategies, skills training, goal setting, and information and education. These common features in apps support several BCTs, including goal setting, reviewing behavioral goals, providing guidance, self-monitoring, and performance feedback. Consequently, these BCTs were the most frequently found among the analyzed apps. An examination of PA and nutrition apps showed that approximately 55% of the analyzed apps included BCTs such as “plan social support” and “provide opportunities for social comparison” [57]. Based on our analysis, we found that only approximately 37% (13/35) of the apps included the BCT “plan social support,” and 29% (10/35) of the apps included the BCT “provide opportunities for social comparison.” Notably, social support is considered an important feature of web-based interventions [58]. As previously mentioned, the inclusion of sharing and app community features is less common among the apps we have examined. This reduced presence may result in decreased user interactivity and limited social support. User interaction enables individuals to share their weight loss progress and strategies within the app, facilitating feedback and suggestions from others. App users can join the community to connect with fellow users, exchange experiences and strategies, and receive valuable support and encouragement.

The fundamental attributes of mobile interventions encompass interactivity, adaptability, time sensitivity, and intraindividual dynamics [59]. Incorporating dynamic elements, such as the timing of information delivery, feedback, and reminders, and personalizing tasks and goals according to individual progress and capabilities align with persuasive techniques and are likely crucial components of an effective, targeted mobile intervention [59-63]. Hence, with the ever-expanding mobile app market, it is imperative to foster increased collaboration between app developers, nutritionists, health care professionals, and behavior change experts. This collaboration could further promote the integration of BCTs into apps, potentially unlocking new opportunities for health promotion.

### Correlation

Our findings unveiled a connection between the quality of various domains within weight management apps and the number of BCTs as well as the quantity of app features. Notably, we observed a positive correlation between the overall app quality and the integration of both technical app features and BCTs. As the specific quality of a given weight management app, both subjectively and objectively, improves, there is a corresponding increase in the inclusion of BCTs and features within the app. Notably, the study revealed a positive correlation between the number of identified BCTs in the analyzed apps and the MARS score across all domains, with the exception of functionality. These findings align with previous studies conducted in various domains. For instance, a study focusing on diet, PA, and sedentary behavior in children and adolescents [43] revealed a positive association between the number of included BCTs and the total MARS score, MARS engagement score, and information quality score. Similarly, in the context of weight management [28], the number of incorporated techniques showed a positive correlation with the overall MARS score, engagement, and aesthetics. Furthermore, in the case of the Mediterranean diet [27], the number of BCTs was positively correlated with the app’s information quality score, overall mean MARS score, subjective quality score, and app-specific quality score. Additionally, in our study, we observed a positive association between the number of features in the apps and app objective quality, subjective quality, and each of the domains. This aligns with findings from the China PA apps study [53]. We also found that the number of app features was positively linked to the number of BCTs, which is consistent with the study conducted on diet, PA, and sedentary behavior in children and adolescents [43].

Our research provides theoretical support for the notion that weight management apps with a greater number of functions and BCTs tend to exhibit higher objective quality. Moreover, it underscores the positive influence of these app features and BCTs on users’ likelihood to recommend the app to others, frequency of use, willingness to pay, and their overall evaluation of the app’s subjective quality. Additionally, the app’s functions and BCTs enhance users’ knowledge, awareness, and attitudes toward weight management. They also strengthen users’ intentions for weight management, increase the likelihood of behavioral changes, and encourage users to seek further help for specific weight management needs. However, it is important to note that our study did not collect data on users’ usage frequency; duration of engagement with weight management apps; willingness to pay; and users’ knowledge, awareness, attitudes, intentions to enhance weight management, and actual behavioral changes resulting from app use. Therefore, caution must be exercised when interpreting our conclusions. This suggests that developers of weight management apps should consider incorporating more detailed, specific, and objective app usage statistics along with subjective user reviews in the future. These data can be invaluable in understanding how users engage with the app, their opinions, and the reasons behind their choices. Such insights can help developers address existing issues, tailor solutions to users’ needs, and enhance overall app quality.

### Strengths and Limitations

To ensure the representation and comprehensive coverage of the apps analyzed in this study, we selected 9 of the most popular Chinese mobile app markets from Qimai Data. China has the largest obese population in the world [4]. However, it is important to note that this study did not evaluate the quality of weight management apps. Instead, we used the validated MARS to assess the quality and features of apps with ratings higher than 4. Additionally, we identified BCTs using an established taxonomy.

This study has some limitations. First, given the rapid development of new apps and potential changes in the content of existing ones, the results presented can be seen as a snapshot of the current state of the included apps. Moreover, it is essential to acknowledge that the apps were assessed after relatively short-term use, which may have concealed certain characteristics, such as those BCTs requiring extended use (eg, follow-up prompts). Second, in alignment with prior research [28], only apps with free content and those with a user score of ≥4 out of 5 were considered for this review. However, it is important to acknowledge that the exclusion of many apps from this review could be considered a limitation, as our findings may not be generalizable to apps that require an immediate paid subscription or apps with a user score of <4 out of 5. Third, it is essential to recognize that the MARS is subject to subjective influences. Factors such as apps requiring users to watch advertisements or the personal preferences of the rater, such as disliking PA while rating an app focused on PA for weight loss, could potentially have a negative impact on scores such as app subjective quality. Furthermore, it is important to note that in this study, weight management apps were assessed by professionals, and this evaluation may not entirely reflect the perspectives of real users of these apps. Therefore, additional research is warranted to obtain user evaluations of weight management apps. Fourth, it is worth noting that, as the Chinese version of the MARS is in the process of localization by other researchers, our study relied on the English version of the MARS for evaluation. This choice may have had an impact on the scoring results, even though the research team consisted of individuals with extensive experience in nutritional management, health behavioral change, and professional dietitians experienced in app development. Fifth, raters in this study used smartphones belonging to other raters for app evaluation, which could have resulted in some degree of unfamiliarity with the devices. Although efforts were made to acquaint the raters with the apps on these phones, there may still be some residual unfamiliarity.

### Conclusions

Publicly available commercial Chinese weight management apps with a user rating of ≥4 exhibited a moderate overall quality. These apps ranked highest in functionality, had moderate scores for aesthetics and engagement, and scored lowest in information quality. The majority of the identified apps incorporated BCTs, with the most frequently used BCTs being goal setting, review of behavioral goals, providing guidance, self-monitoring, and performance feedback. Apps of higher quality typically included a greater number of technical app features and BCTs. There is a need for further efforts in the future to enhance these apps to engage users effectively. Efforts to boost user-app interaction and provide more comprehensive, evidence-based information within apps are crucial. Further research is necessary to identify the optimal number and combination of features and BCTs that can effectively influence behavioral change in weight management.

## Abbreviations

BCT: behavior change technique
KR-20: Kuder-Richardson index 20
MARS: Mobile Application Rating Scale
mHealth: mobile health
PA: physical activity
PRISMA: Preferred Reporting Items for Systematic Reviews and Meta-Analyses
